# Supplementary figures and images for: Evaluation of first and second trimester maternal thyroid profile on the prediction of gestational diabetes mellitus and post load glycemia
Source: PLoS One. 2023 Jan 13;18(1):e0280513. doi: 10.1371/journal.pone.0280513 (PMC9838876; doi:10.1371/journal.pone.0280513)

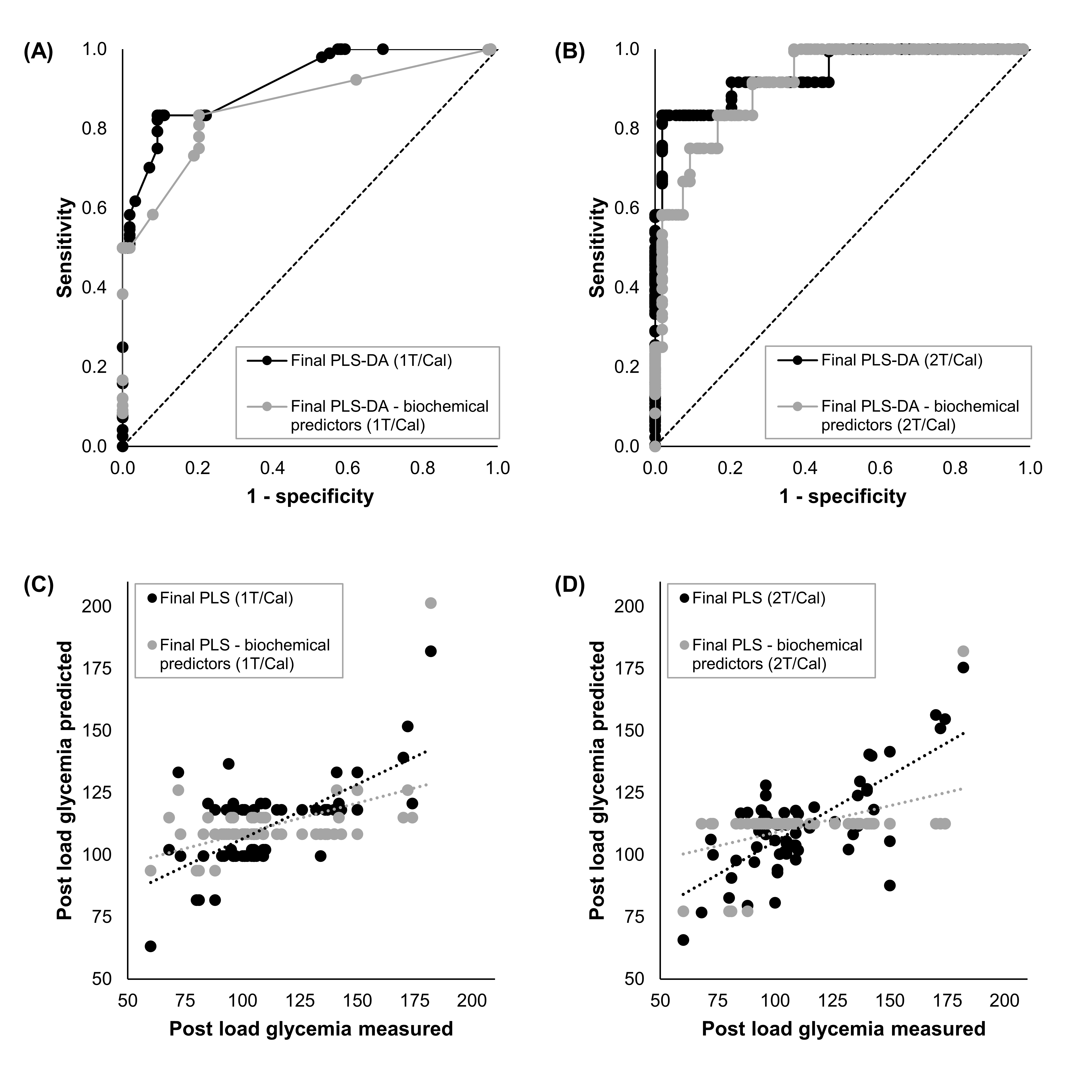

Supplement: S1 Fig — A-B) For the prediction of GDM in the first (A) or second (B) trimester of pregnancy by PLS-DA. C-D) For the prediction of post load glycemia in the first (C) or second (D) trimester of pregnancy by PLS. The four plots show the calibration performance of the final models with their full set of predictors (black) and after the remotion of biochemical markers (grey). 1T: First trimester. 2T: Second trimester. PLS-DA: Partial least squares discriminant analysis. PLS: Partial least squares. CV: Cross-validation. (TIF) [file pone.0280513.s001.tif]

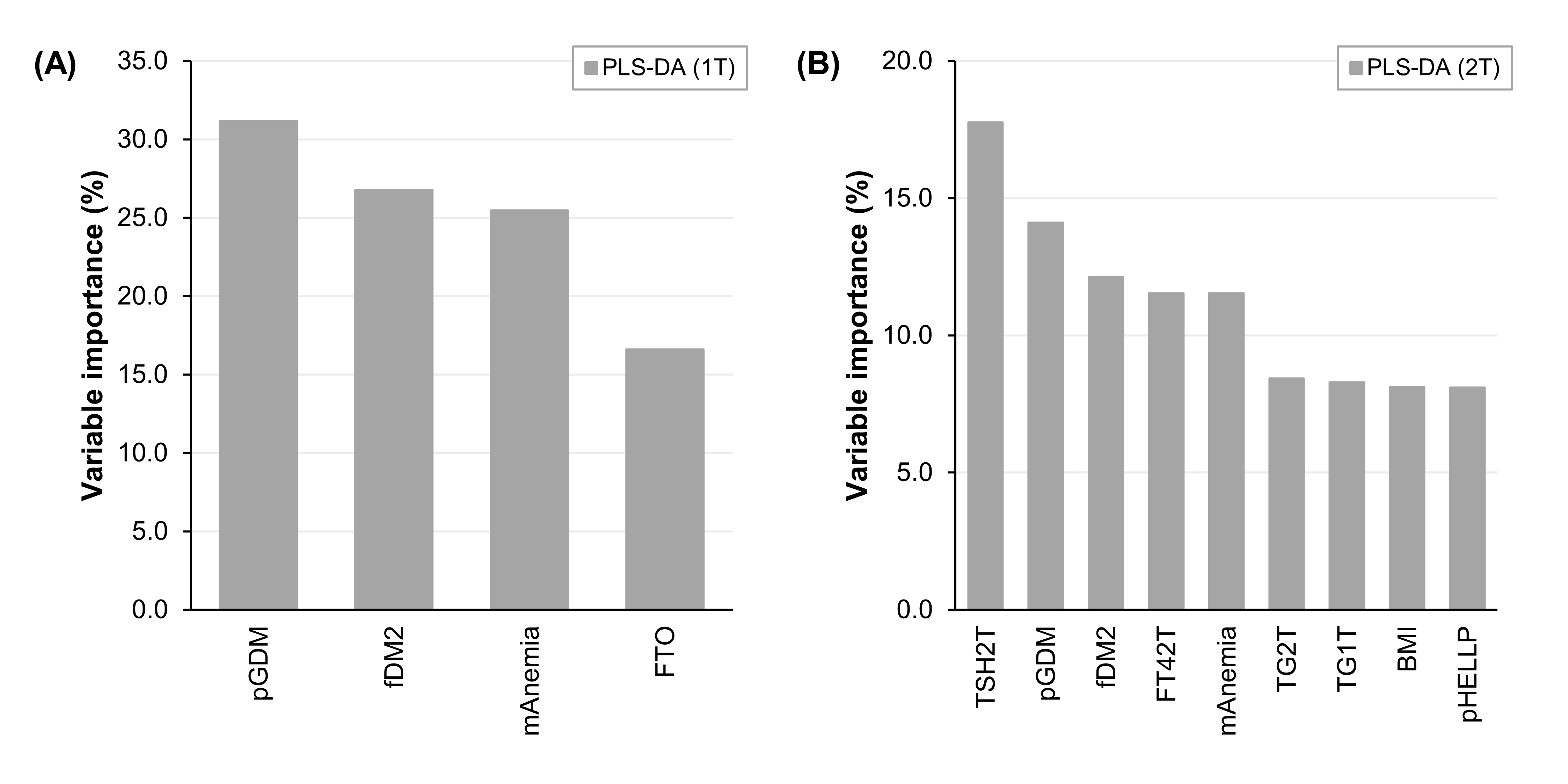

Supplement: S2 Fig — (A) Built on first trimester data only. (B) Including second trimester data. 1T: First trimester. 2T: Second trimester. PLS-DA: Partial least squares discriminant analysis. pGDM: Prior gestational diabetes mellitus. fDM2: Family history of type 2 diabetes. mAnemia: Personal anemia. FTO: Fat mass and obesity-associated genotype (rs9939609). TSH: Thyroid stimulating hormone. FT4: Free thyroxine. TG: Thyroglobulin. BMI: Body mass index. pHELLP: Prior hemolysis elevated liver enzymes and low platelets syndrome. (TIF) [file pone.0280513.s002.tif]

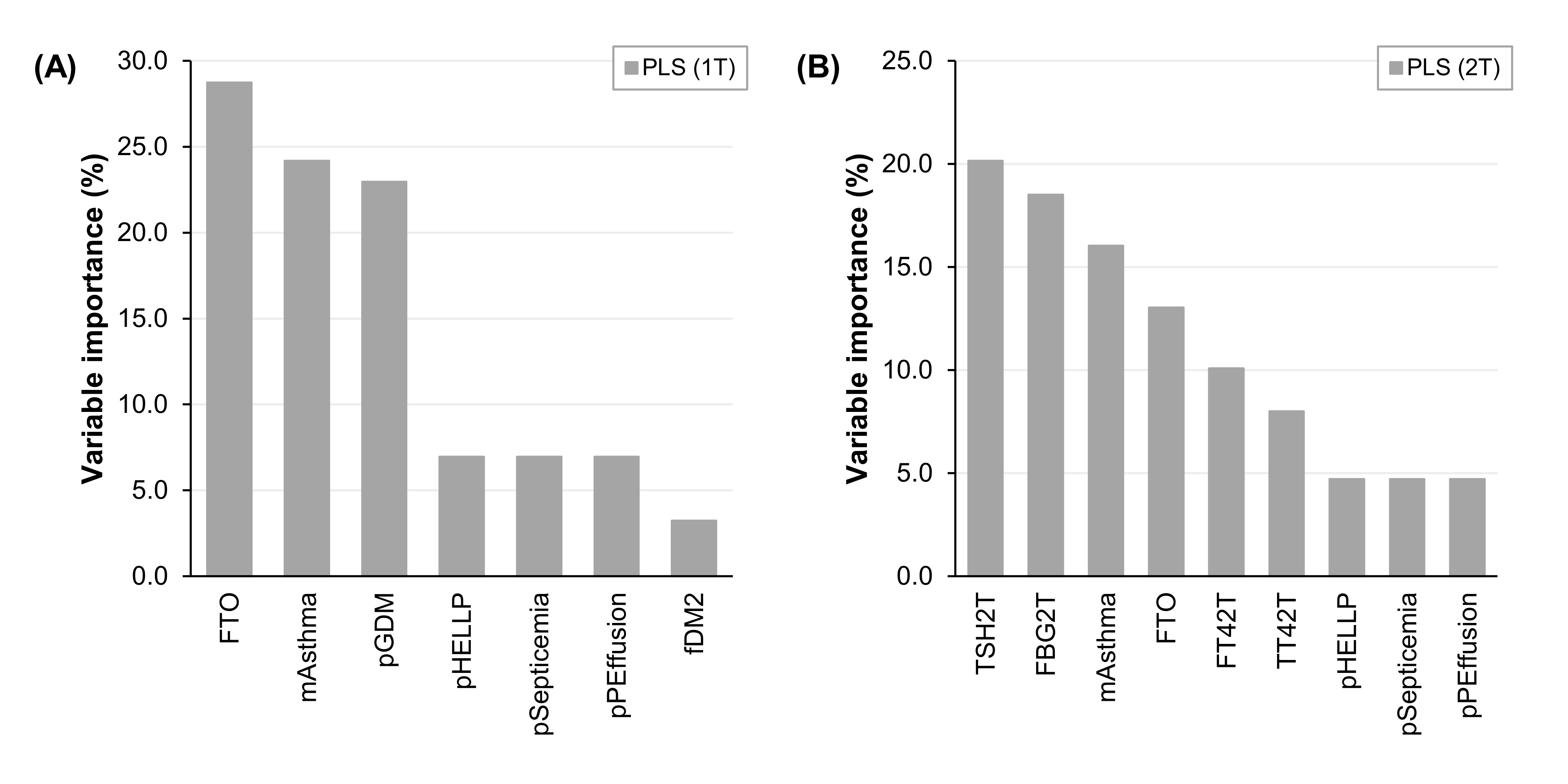

Supplement: S3 Fig — (A) Built on first trimester data only. (B) Including second trimester data. 1T: First trimester. 2T: Second trimester. PLS: Partial least squares. FTO: Fat mass and obesity-associated genotype (rs9939609). mAsthma: Personal asthma. pGDM: Prior gestational diabetes mellitus. pHELLP: Prior hemolysis elevated liver enzymes and low platelets syndrome. pSepticemia: Prior septicemia. pPEffusion: Prior pleural effusion. fDM2: Family history of type 2 diabetes. TSH: Thyroid stimulating hormone. FBG: Fasting blood glucose. FT4: Free thyroxine. TT4: Total thyroxine. (TIF) [file pone.0280513.s003.tif]
