# Supplementary material for: Evaluation of first and second trimester maternal thyroid profile on the prediction of gestational diabetes mellitus and post load glycemia
Source: PLoS One. 2023 Jan 13;18(1):e0280513. doi: 10.1371/journal.pone.0280513 (PMC9838876; doi:10.1371/journal.pone.0280513)
Supplement: S1 Table — NGT: Normal glucose tolerance. GDM: Gestational diabetes mellitus. BMI: Body mass index. HELLP: Hemolysis elevated liver enzymes and low platelets syndrome. IUGR: Intrauterine growth restriction. PCOS: Polycystic ovary syndrome. DM1: Type 1 diabetes. DM2: Type 2 diabetes. FTO: Fat mass and obesity-associated gene. TSH: Thyroid stimulating hormone. TT3: Total triiodothyronine. TT4: Total thyroxine. FT4: Free T4. TG: Thyroglobulin. aTG: TG antibody. aTPO thyroid peroxidase antibody. TRAb: TSH receptor antibody. OGTT: Oral glucose tolerance test. Qualitative variables are presented as percentage (proportion); quantitative variables with normal distribution, as mean ± standard deviation; and quantitative variables with non-normal distribution, as median (interquartile range). NS: Not significant. * p<0.05. ** p<0.01. **** p<0.0001. (DOCX) [file pone.0280513.s004.docx]

| **Variable** | **Unit** | **NGT (n=54)** | **GDM (n=12)** | **p value** | | **All (n=66)** |
| --- | --- | --- | --- | --- | --- | --- |
| **First trimester** | | | | | | |
| Age | years | 30 ± 5 | 30 ± 7 | 0.724 | NS | 30 ± 6 |
| Height | m | 1.60 ± 0.06 | 1.60 ± 0.05 | 0.804 | NS | 1.60 ± 0.06 |
| Weight | Kg | 70.0 (60.4-77.1) | 77.0 (66.0-81.8) | 0.084 | NS | 70.5 (61.9-78.2) |
| BMI | Kg/m^2^ | 27.4 (23.5-31.1) | 30.3 (27.1-31.6) | 0.046 | * | 27.8 (24.5-31.2) |
| Systolic pressure | mmHg | 109 ± 11 | 116 ± 7 | 0.042 | * | 110 ± 11 |
| Diastolic pressure | mmHg | 67 ± 7 | 66 ± 6 | 0.443 | NS | 67 ± 7 |
| Supplement consumption | % | 57.4 (31/54) | 41.7 (5/12) | 0.355 | NS | 54.5 (36/66) |
| Hyperemesis | % | 24.1 (13/54) | 16.7 (2/12) | 0.719 | NS | 22.7 (15/66) |
| Vaginal bleeding | % | 7.4 (4/54) | 16.7 (2/12) | 0.298 | NS | 9.1 (6/66) |
| Drug use at pregnancy | % |  |  |  |  |  |
| *Cigarette* |  | 3.7 (2/54) | 0.0 (0/12) | >0.999 | NS | 3.0 (2/66) |
| *Alcohol* |  | 1.9 (1/54) | 0.0 (0/12) | >0.999 | NS | 1.5 (1/66) |
| *Other drugs* |  | 3.7 (2/54) | 0.0 (0/12) | >0.999 | NS | 3.0 (2/66) |
| Fasting glycemia | mg/dL | 81 ± 8 | 83 ± 4 | 0.272 | NS | 81 ± 7 |
| TSH | µIU/mL | 1.41 (1.03-2.36) | 1.58 (1.01-2.40) | 0.752 | NS | 1.41 (1.02-2.36) |
| TT3 | ng/mL | 1.62 (1.55-1.80) | 1.61 (1.54-1.85) | 0.789 | NS | 1.62 (1.55-1.82) |
| TT4 | µg/dL | 11.30 (10.55-12.50) | 10.90 (9.57-12.80) | 0.436 | NS | 11.30 (10.40-12.50) |
| FT4 | ng/dL | 0.99 (0.85-1.11) | 0.99 (0.89-1.08) | 0.905 | NS | 0.99 (0.86-1.11) |
| TG | ng/mL | 16.69 (9.70-18.13) | 17.63 (9.90-22.17) | 0.394 | NS | 16.69 (9.74-19.14) |
| aTG | IU/mL | 9.71 (5.37-11.99) | 12.83 (6.12-16.89) | 0.239 | NS | 10.09 (5.40-13.29) |
| aTPO | IU/mL | 1.32 (0.43-3.51) | 1.22 (0.38-1.71) | 0.407 | NS | 1.32 (0.43-2.51) |
| TRAb | IU/L | 0.25 (0.25-0.29) | 0.25 (0.25-0.43) | 0.338 | NS | 0.25 (0.25-0.32) |
| Preconception data | | | | | | |
| Drug use before pregnancy | % |  |  |  |  |  |
| *Cigarette* |  | 37.0 (20/54) | 33.3 (4/12) | >0.999 | NS | 36.4 (24/66) |
| *Alcohol* |  | 57.4 (31/54) | 50.0 (6/12) | 0.752 | NS | 56.1 (37/66) |
| *Other drugs* |  | 20.4 (11/54) | 0.0 (0/12) | 0.193 | NS | 16.7 (11/66) |
| Prior pregnancy issues | % |  |  |  |  |  |
| *GDM* |  | 1.9 (1/54) | 33.3 (4/12) | 0.003 | ** | 7.6 (5/66) |
| *Hypertension* |  | 1.9 (1/54) | 0.0 (0/12) | >0.999 | NS | 1.5 (1/66) |
| *Preeclampsia* |  | 1.9 (1/54) | 0.0 (0/12) | >0.999 | NS | 1.5 (1/66) |
| *HELLP* |  | 0.0 (0/54) | 8.3 (1/12) | 0.182 | NS | 1.5 (1/66) |
| *IUGR* |  | 1.9 (1/54) | 0.0 (0/12) | >0.999 | NS | 1.5 (1/66) |
| *Preterm birth* |  | 3.7 (2/54) | 8.3 (1/12) | 0.458 | NS | 4.5 (3/66) |
| *Septicemia* |  | 0.0 (0/54) | 8.3 (1/12) | 0.182 | NS | 1.5 (1/66) |
| *Pleural effusion* |  | 0.0 (0/54) | 8.3 (1/12) | 0.182 | NS | 1.5 (1/66) |
| *Fetal arrhythmia* |  | 1.9 (1/54) | 0.0 (0/12) | >0.999 | NS | 1.5 (1/66) |
| *Hernia* |  | 1.9 (1/54) | 0.0 (0/12) | >0.999 | NS | 1.5 (1/66) |
| Prior non-viable pregnancy | % | 18.5 (10/54) | 8.3 (1/12) | 0.673 | NS | 16.7 (11/66) |
| Fertility problems | % | 14.8 (8/54) | 0.0 (0/12) | 0.333 | NS | 12.1 (8/66) |
| PCOS | % | 24.1 (13/54) | 16.7 (2/12) | 0.719 | NS | 22.7 (15/66) |
| First period age | years | 13 (12-14) | 12 (11-13) | 0.095 | NS | 13 (12-14) |
| Last period month | % |  |  | 0.699 | NS |  |
| *January* |  | 3.7 (2/54) | 0.0 (0/12) |  |  | 3.0 (2/66) |
| *February* |  | 5.6 (3/54) | 25.0 (3/12) |  |  | 9.1 (6/66) |
| *March* |  | 9.3 (5/54) | 0.0 (0/12) |  |  | 7.6 (5/66) |
| *April* |  | 9.3 (5/54) | 8.3 (1/12) |  |  | 9.1 (6/66) |
| *May* |  | 11.1 (6/54) | 8.3 (1/12) |  |  | 10.6 (7/66) |
| *June* |  | 13.0 (7/54) | 8.3 (1/12) |  |  | 12.1 (8/66) |
| *July* |  | 11.1 (6/54) | 16.7 (2/12) |  |  | 12.1 (8/66) |
| *August* |  | 9.3 (5/54) | 0.0 (0/12) |  |  | 7.6 (5/66) |
| *September* |  | 3.7 (2/54) | 16.7 (2/12) |  |  | 6.1 (4/66) |
| *October* |  | 11.1 (6/54) | 8.3 (1/12) |  |  | 10.6 (7/66) |
| *November* |  | 11.1 (6/54) | 8.3 (1/12) |  |  | 10.6 (7/66) |
| *December* |  | 1.9 (1/54) | 0.0 (0/12) |  |  | 1.5 (1/66) |
| Personal morbid history | % |  |  |  |  |  |
| *Insulin resistance* |  | 3.7 (2/54) | 0.0 (0/12) | >0.999 | NS | 3.0 (2/66) |
| *Hypertension* |  | 1.9 (1/54) | 0.0 (0/12) | >0.999 | NS | 1.5 (1/66) |
| *Thyroid disfunction* |  | 7.4 (4/54) | 0.0 (0/12) | >0.999 | NS | 6.1 (4/66) |
| *Anemia* |  | 0.0 (0/54) | 16.7 (2/12) | 0.031 | * | 3.0 (2/66) |
| *Asthma* |  | 7.4 (4/54) | 0.0 (0/12) | >0.999 | NS | 6.1 (4/66) |
| *Rhinitis* |  | 1.9 (1/54) | 0.0 (0/12) | >0.999 | NS | 1.5 (1/66) |
| *Osteopenia* |  | 1.9 (1/54) | 0.0 (0/12) | >0.999 | NS | 1.5 (1/66) |
| *Von Willebrand* |  | 1.9 (1/54) | 0.0 (0/12) | >0.999 | NS | 1.5 (1/66) |
| *Neoplasia* |  | 0.0 (0/54) | 8.3 (1/12) | 0.182 | NS | 1.5 (1/66) |
| Family morbid history | % |  |  |  |  |  |
| *Insulin resistance* |  | 3.7 (2/54) | 8.3 (1/12) | 0.458 | NS | 4.5 (3/66) |
| *Prediabetes* |  | 1.9 (1/54) | 0.0 (0/12) | >0.999 | NS | 1.5 (1/66) |
| *DM1* |  | 1.9 (1/54) | 0.0 (0/12) | >0.999 | NS | 1.5 (1/66) |
| *DM2* |  | 20.4 (11/54) | 66.7 (8/12) | 0.003 | ** | 28.8 (19/66) |
| *Hypertension* |  | 33.3 (18/54) | 66.7 (8/12) | 0.049 | * | 39.4 (26/66) |
| *Thrombosis* |  | 1.9 (1/54) | 0.0 (0/12) | >0.999 | NS | 1.5 (1/66) |
| *Heart surgery* |  | 1.9 (1/54) | 0.0 (0/12) | >0.999 | NS | 1.5 (1/66) |
| *Myocardial infarction* |  | 1.9 (1/54) | 0.0 (0/12) | >0.999 | NS | 1.5 (1/66) |
| *Cerebrovascular accident* |  | 1.9 (1/54) | 0.0 (0/12) | >0.999 | NS | 1.5 (1/66) |
| *Hypothyroidism* |  | 16.7 (9/54) | 25.0 (3/12) | 0.679 | NS | 18.2 (12/66) |
| *Hyperthyroidism* |  | 5.6 (3/54) | 16.7 (2/12) | 0.222 | NS | 7.6 (5/66) |
| *Gout* |  | 1.9 (1/54) | 0.0 (0/12) | >0.999 | NS | 1.5 (1/66) |
| *Chronic kidney disease* |  | 1.9 (1/54) | 0.0 (0/12) | >0.999 | NS | 1.5 (1/66) |
| *Rheumatoid arthritis* |  | 0.0 (0/54) | 8.3 (1/12) | 0.182 | NS | 1.5 (1/66) |
| *Asthma* |  | 9.3 (5/54) | 0.0 (0/12) | 0.575 | NS | 7.6 (5/66) |
| *Neoplasia* |  | 1.9 (1/54) | 0.0 (0/12) | >0.999 | NS | 1.5 (1/66) |
| FTO genotype (rs9939609) | % |  |  | 0.047 | * |  |
| *TT* |  | 53.7 (29/54) | 25.0 (3/12) |  |  | 48.5 (32/66) |
| *TA* |  | 44.4 (24/54) | 66.7 (8/12) |  |  | 48.5 (32/66) |
| *AA* |  | 1.9 (1/54) | 8.3 (1/12) |  |  | 3.0 (2/66) |
| **Second trimester** | | | | | | |
| TSH | µIU/mL | 1.42 (1.10-2.09) | 3.25 (2.28-4.38) | 0.001 | ** | 1.54 (1.10-2.53) |
| TT3 | ng/mL | 1.92 (1.90-2.04) | 2.02 (1.92-2.36) | 0.027 | * | 1.92 (1.90-2.11) |
| TT4 | µg/dL | 12.90 (12.59-13.33) | 11.15 (10.63-13.30) | 0.088 | NS | 12.90 (11.35-13.33) |
| FT4 | ng/dL | 0.79 (0.76-0.81) | 0.68 (0.63-0.78) | 0.006 | ** | 0.77 (0.76-0.81) |
| TG | ng/mL | 17.37 (11.92-20.91) | 21.44 (13.63-23.88) | 0.067 | NS | 18.81 (12.28-20.96) |
| aTG | IU/mL | 4.50 (4.30-7.05) | 16.93 (8.42-18.45) | 0.020 | * | 4.89 (4.31-16.85) |
| aTPO | IU/mL | 1.18 (1.09-1.40) | 1.22 (1.11-2.05) | 0.280 | NS | 1.18 (1.09-1.41) |
| TRAb | IU/L | 0.25 (0.25-0.52) | 0.25 (0.25-0.25) | 0.228 | NS | 0.25 (0.25-0.38) |
| Fasting glycemia | mg/dL | 79 ± 8 | 86 ± 10 | 0.013 | * | 80 ± 9 |
| OGTT glycemia (75 g, 2 h) | mg/dL | 102 (94-109) | 150 (141-172) | <0.001 | **** | 105 (96-134) |
