# Supplementary material for: Evaluation of first and second trimester maternal thyroid profile on the prediction of gestational diabetes mellitus and post load glycemia
Source: PLoS One. 2023 Jan 13;18(1):e0280513. doi: 10.1371/journal.pone.0280513 (PMC9838876; doi:10.1371/journal.pone.0280513)
Supplement: S2 Table — TSH: Thyroid stimulating hormone. TT3: Total triiodothyronine. FT4: Free thyroxine. aTG: Thyroglobulin antibody. Quantitative variables with non-normal distribution are presented as median (interquartile range). NS: Not significant. * p<0.05. ** p<0.01. *** p<0.001. **** p<0.0001. (DOCX) [file pone.0280513.s005.docx]

**(A) Full cohort (n=66)**

| **Variable** | **Unit** | **First trimester** | **Second trimester** | **p value** |  |
| --- | --- | --- | --- | --- | --- |
| TSH | µIU/mL | 1.41 (1.02-2.36) | 1.54 (1.10-2.53) | 0.798 | NS |
| TT3 | ng/mL | 1.62 (1.55-1.82) | 1.92 (1.90-2.11) | <0.001 | **** |
| FT4 | ng/dL | 0.99 (0.86-1.11) | 0.77 (0.76-0.81) | <0.001 | **** |
| aTG | IU/mL | 10.09 (5.40-13.29) | 4.89 (4.31-16.85) | 0.030 | * |

**(B) Normal glucose tolerance (n=54)**

| **Variable** | **Unit** | **First trimester** | **Second trimester** | **p value** |  |
| --- | --- | --- | --- | --- | --- |
| TSH | µIU/mL | 1.41 (1.03-2.36) | 1.42 (1.10-2.09) | 0.458 | NS |
| TT3 | ng/mL | 1.62 (1.55-1.80) | 1.92 (1.90-2.04) | <0.001 | **** |
| FT4 | ng/dL | 0.99 (0.85-1.11) | 0.79 (0.76-0.81) | <0.001 | **** |
| aTG | IU/mL | 9.71 (5.37-11.99) | 4.50 (4.30-7.05) | 0.010 | ** |

**(C) Gestational diabetes mellitus (n=12)**

| **Variable** | **Unit** | **First trimester** | **Second trimester** | **p value** |  |
| --- | --- | --- | --- | --- | --- |
| TSH | µIU/mL | 1.58 (1.01-2.40) | 3.25 (2.28-4.38) | 0.067 | NS |
| TT3 | ng/mL | 1.61 (1.54-1.85) | 2.02 (1.92-2.36) | 0.005 | ** |
| FT4 | ng/dL | 0.99 (0.89-1.08) | 0.68 (0.63-0.78) | 0.001 | *** |
| aTG | IU/mL | 12.83 (6.12-16.89) | 16.93 (8.42-18.45) | 0.966 | NS |
