# Supplementary material for: Evaluation of first and second trimester maternal thyroid profile on the prediction of gestational diabetes mellitus and post load glycemia
Source: PLoS One. 2023 Jan 13;18(1):e0280513. doi: 10.1371/journal.pone.0280513 (PMC9838876; doi:10.1371/journal.pone.0280513)
Supplement: S3 Table — a Model with the highest cross-validation non-error rate (CV-NER) for GDM prediction, using 1T data only. b Model with the highest CV-NER for GDM prediction, including 2T data. LR: Logistic regression. L-SVM: Linear support vector machine. PLS-DA: Partial least squares discriminant analysis. CART: Classification and regression tree. XGB: Extreme gradient boosting. 1T: First trimester. 2T: Second trimester. Thy: Thyroid predictors. NoThy: Non-thyroid predictors. (DOCX) [file pone.0280513.s006.docx]

| **Parameter** | **Maternal predictors** | **Calibration** | | | | | **Cross-validation** | | | | |
| --- | --- | --- | --- | --- | --- | --- | --- | --- | --- | --- | --- |
|  |  | **LR** | **L-SVM** | **PLS-DA** | **CART** | **XGB** | **LR** | **L-SVM** | **PLS-DA** | **CART** | **XGB** |
| Sensitivity (%) | Thy1T | 16.7 | 8.3 | 33.3 | 0.0 | 50.0 | 8.3 | 0.0 | 16.7 | 0.0 | 8.3 |
|  | Thy2T | 75.0 | 66.7 | 75.0 | 58.3 | 100.0 | 58.3 | 50.0 | 75.0 | 58.3 | 66.7 |
|  | Thy1T + Thy2T | 91.7 | 58.3 | 91.7 | 58.3 | 100.0 | 33.3 | 50.0 | 75.0 | 58.3 | 66.7 |
|  | NoThy1T | 100.0 | 66.7 | 91.7 | 58.3 | 100.0 | 66.7 | 50.0 | 75.0 | 50.0 | 25.0 |
|  | NoThy1T + Thy1T | 100.0 | 83.3 | 100.0 ^a^ | 33.3 | 100.0 | 66.7 | 50.0 | 75.0 ^a^ | 25.0 | 25.0 |
|  | NoThy1T + Thy2T | 100.0 | 83.3 | 83.3 | 58.3 | 100.0 | 66.7 | 66.7 | 83.3 | 58.3 | 58.3 |
|  | NoThy1T + Thy1T + Thy2T | 100.0 | 75.0 | 91.7 ^b^ | 58.3 | 100.0 | 66.7 | 66.7 | 83.3 ^b^ | 58.3 | 58.3 |
|  | NoThy2T | 8.3 | 0.0 | 58.3 | 0.0 | 0.0 | 8.3 | 0.0 | 50.0 | 0.0 | 0.0 |
|  | NoThy2T + Thy1T | 25.0 | 8.3 | 33.3 | 0.0 | 16.7 | 16.7 | 8.3 | 16.7 | 0.0 | 0.0 |
|  | NoThy2T + Thy2T | 83.3 | 75.0 | 83.3 | 58.3 | 100.0 | 58.3 | 66.7 | 75.0 | 58.3 | 66.7 |
|  | NoThy2T + Thy1T + Thy2T | 91.7 | 75.0 | 91.7 | 58.3 | 100.0 | 50.0 | 58.3 | 83.3 | 58.3 | 66.7 |
|  | NoThy1T + NoThy2T | 100.0 | 91.7 | 83.3 | 58.3 | 100.0 | 66.7 | 58.3 | 83.3 | 33.3 | 33.3 |
|  | NoThy1T + NoThy2T + Thy1T | 100.0 | 83.3 | 91.7 | 33.3 | 100.0 | 66.7 | 66.7 | 75.0 | 25.0 | 25.0 |
|  | NoThy1T + NoThy2T + Thy2T | 100.0 | 83.3 | 83.3 | 58.3 | 100.0 | 58.3 | 66.7 | 83.3 | 58.3 | 58.3 |
|  | NoThy1T + NoThy2T + Thy1T + Thy2T | 100.0 | 91.7 | 91.7 | 58.3 | 100.0 | 58.3 | 66.7 | 83.3 | 58.3 | 58.3 |
| Specificity (%) | Thy1T | 98.1 | 100.0 | 90.7 | 100.0 | 100.0 | 94.4 | 100.0 | 88.9 | 100.0 | 96.3 |
|  | Thy2T | 98.1 | 100.0 | 92.6 | 100.0 | 100.0 | 96.3 | 98.1 | 88.9 | 100.0 | 98.1 |
|  | Thy1T + Thy2T | 100.0 | 100.0 | 94.4 | 100.0 | 100.0 | 85.2 | 98.1 | 87.0 | 100.0 | 100.0 |
|  | NoThy1T | 100.0 | 100.0 | 94.4 | 98.1 | 100.0 | 83.3 | 96.3 | 85.2 | 96.3 | 94.4 |
|  | NoThy1T + Thy1T | 100.0 | 100.0 | 100.0 ^a^ | 98.1 | 100.0 | 85.2 | 96.3 | 87.0 ^a^ | 98.1 | 98.1 |
|  | NoThy1T + Thy2T | 100.0 | 100.0 | 94.4 | 100.0 | 100.0 | 81.5 | 100.0 | 88.9 | 100.0 | 100.0 |
|  | NoThy1T + Thy1T + Thy2T | 100.0 | 100.0 | 94.4 ^b^ | 100.0 | 100.0 | 83.3 | 100.0 | 90.7 ^b^ | 100.0 | 100.0 |
|  | NoThy2T | 100.0 | 100.0 | 79.6 | 100.0 | 100.0 | 100.0 | 100.0 | 79.6 | 100.0 | 100.0 |
|  | NoThy2T + Thy1T | 100.0 | 100.0 | 83.3 | 100.0 | 100.0 | 92.6 | 100.0 | 79.6 | 100.0 | 98.1 |
|  | NoThy2T + Thy2T | 100.0 | 100.0 | 88.9 | 100.0 | 100.0 | 96.3 | 100.0 | 87.0 | 100.0 | 100.0 |
|  | NoThy2T + Thy1T + Thy2T | 100.0 | 100.0 | 92.6 | 100.0 | 100.0 | 81.5 | 98.1 | 85.2 | 100.0 | 100.0 |
|  | NoThy1T + NoThy2T | 100.0 | 100.0 | 96.3 | 98.1 | 100.0 | 85.2 | 96.3 | 87.0 | 98.1 | 92.6 |
|  | NoThy1T + NoThy2T + Thy1T | 100.0 | 100.0 | 96.3 | 98.1 | 100.0 | 79.6 | 94.4 | 83.3 | 98.1 | 96.3 |
|  | NoThy1T + NoThy2T + Thy2T | 100.0 | 100.0 | 94.4 | 100.0 | 100.0 | 79.6 | 100.0 | 88.9 | 100.0 | 100.0 |
|  | NoThy1T + NoThy2T + Thy1T + Thy2T | 100.0 | 100.0 | 94.4 | 100.0 | 100.0 | 75.9 | 100.0 | 88.9 | 100.0 | 100.0 |
