# Supplementary material for: Evaluation of first and second trimester maternal thyroid profile on the prediction of gestational diabetes mellitus and post load glycemia
Source: PLoS One. 2023 Jan 13;18(1):e0280513. doi: 10.1371/journal.pone.0280513 (PMC9838876; doi:10.1371/journal.pone.0280513)
Supplement: S4 Table — a Model with the lowers RE for GDM prediction, using 1T data only. b Model with the lowest RE for GDM prediction, including 2T data. PLS: Partial least squares. RMSEC: Root mean square error of calibration. RMSECV: Root mean square error of cross-validation. 1T: First trimester. 2T: Second trimester. Thy: Thyroid predictors. NoThy: Non-thyroid predictors. (DOCX) [file pone.0280513.s007.docx]

| **Maternal predictors** | **PLS** | |
| --- | --- | --- |
|  | **Calibration RMSE** | **Cross-validation RMSE** |
| Thy1T | 24.9 | 27.5 |
| Thy2T | 18.1 | 22.4 |
| Thy1T + Thy2T | 19.8 | 25.6 |
| NoThy1T | 18.1 ^a^ | 23.8 ^a^ |
| NoThy1T + Thy1T | 17.5 | 24.1 |
| NoThy1T + Thy2T | 17.0 | 21.3 |
| NoThy1T + Thy1T + Thy2T | 16.5 | 22.3 |
| NoThy2T | 24.1 | 25.0 |
| NoThy2T + Thy1T | 23.5 | 26.4 |
| NoThy2T + Thy2T | 19.4 | 22.4 |
| NoThy2T + Thy1T + Thy2T | 19.0 | 24.7 |
| NoThy1T + NoThy2T | 17.3 | 23.3 |
| NoThy1T + NoThy2T + Thy1T | 16.7 | 23.6 |
| NoThy1T + NoThy2T + Thy2T | 16.4 ^b^ | 20.9 ^b^ |
| NoThy1T + NoThy2T + Thy1T + Thy2T | 16.0 | 21.9 |
