# Supplementary material for: Evaluation of first and second trimester maternal thyroid profile on the prediction of gestational diabetes mellitus and post load glycemia
Source: PLoS One. 2023 Jan 13;18(1):e0280513. doi: 10.1371/journal.pone.0280513 (PMC9838876; doi:10.1371/journal.pone.0280513)
Supplement: S5 Table — a For PLS-DA: NoThy1T + Thy1T. For PLS: NoThy1T. b Model with the lowest RE for post load glycemia prediction, using 1T data only. Predictors: FTO, pHELLP, pSepticemia, pPEffusion, mAsthma, fDM2, pGDM. c Model with the highest NER for GDM prediction, using 1T data only. Predictors: mAnemia, FTO, pGDM, fDM2. d For PLS-DA: NoThy1T + Thy1T + Thy2T. For PLS: NoThy1T + NoThy2T + Thy2T. e Model with the highest NER for GDM prediction, including 2T data. Predictors: TSH2T, pGDM, fDM2, FT42T, mAnemia, TG2T, TG1T, BMI, pHELLP. f Model with the lowest RE for post load glycemia prediction, including 2T data. Predictors: TSH2T, FT42T, FBG2T, TT42T, FTO, pHELLP, pSepticemia, pPEffusion, mAsthma. PLS-DA: Partial least squares discriminant analysis. PLS: Partial least squares. Se: Sensitivity. Sp: Specificity. NER: Non-error rate. RMSE: Root mean square error. RE: Relative error. NoThy: Non-thyroid predictors. Thy: Thyroid predictors. 1T: First trimester. 2T: Second trimester. FTO: Fat mass and obesity-associated genotype (rs9939609). pHELLP: Prior hemolysis elevated liver enzymes and low platelets syndrome. pSepticemia: Prior septicemia. pPEffusion: Prior pleural effusion. mAsthma: Personal asthma. fDM2: Family history of type 2 diabetes. pGDM: Prior gestational diabetes mellitus. mAnemia: Personal anemia. TSH: Thyroid stimulating hormone. FT4: Free thyroxine. TG: Thyroglobulin. BMI: Body mass index. FBG: Fasting blood glucose. TT4: Total thyroxine. (DOCX) [file pone.0280513.s008.docx]

| **Maternal predictors** | **Calibration** | | | | | **Cross-validation** | | | | |
| --- | --- | --- | --- | --- | --- | --- | --- | --- | --- | --- |
|  | **PLS-DA** | | | **PLS** | | **PLS-DA** | | | **PLS** | |
|  | **Se (%)** | **Sp (%)** | **NER (%)** | **RMSE** | **RE (%)** | **Se (%)** | **Sp (%)** | **NER (%)** | **RMSE** | **RE (%)** |
| ***First trimester*** | | | | | | | | | | |
| Full ^a^ | 100.0 | 100.0 | 100.0 | 18.1 | 16.3 | 75.0 | 87.0 | 81.0 | 23.8 | 21.4 |
| Top 10 | 75.0 | 94.4 | 84.7 | 21.1 | 19.0 | 75.0 | 88.9 | 81.9 | 23.2 | 20.8 |
| Top 9 | 83.3 | 94.4 | 88.9 | 21.0 | 18.8 | 75.0 | 88.9 | 81.9 | 23.3 | 20.9 |
| Top 8 | 83.3 | 92.6 | 88.0 | 19.4 | 17.4 | 75.0 | 87.0 | 81.0 | 23.2 | 20.8 |
| Top 7 | 83.3 | 90.7 | 87.0 | 19.6 ^b^ | 17.6 ^b^ | 75.0 | 87.0 | 81.0 | 23.1 ^b^ | 20.7 ^b^ |
| Top 6 | 83.3 | 94.4 | 88.9 | 20.9 | 18.7 | 75.0 | 90.7 | 82.9 | 23.2 | 20.8 |
| Top 5 | 83.3 | 90.7 | 87.0 | 21.4 | 19.2 | 66.7 | 90.7 | 78.7 | 23.6 | 21.2 |
| Top 4 | 83.3 ^c^ | 90.7 ^c^ | 87.0 ^c^ | 23.3 | 20.9 | 83.3 ^c^ | 90.7 ^c^ | 87.0 ^c^ | 25.5 | 22.9 |
| Top 3 | 58.3 | 96.3 | 77.3 | 23.3 | 20.9 | 50.0 | 96.3 | 73.1 | 25.5 | 22.9 |
| Top 2 | 25.0 | 98.1 | 61.6 | 23.3 | 20.9 | 25.0 | 98.1 | 61.6 | 25.4 | 22.8 |
| Top 1 | 16.7 | 100.0 | 58.3 | 24.6 | 22.1 | 16.7 | 100.0 | 58.3 | 25.5 | 22.9 |
| ***Second trimester*** | | | | | | | | | | |
| Full ^d^ | 91.7 | 94.4 | 93.1 | 16.4 | 14.7 | 83.3 | 90.7 | 87.0 | 20.9 | 18.7 |
| Top 10 | 83.3 | 98.1 | 90.7 | 17.9 | 16.1 | 83.3 | 96.3 | 89.8 | 20.7 | 18.6 |
| Top 9 | 83.3 ^e^ | 96.3 ^e^ | 89.8 ^e^ | 17.9 ^f^ | 16.1 ^f^ | 83.3 ^e^ | 96.3 ^e^ | 89.8 ^e^ | 20.5 ^f^ | 18.4 ^f^ |
| Top 8 | 83.3 | 94.4 | 88.9 | 18.9 | 17.0 | 83.3 | 94.4 | 88.9 | 21.6 | 19.4 |
| Top 7 | 83.3 | 96.3 | 89.8 | 19.6 | 17.6 | 83.3 | 94.4 | 88.9 | 21.5 | 19.3 |
| Top 6 | 83.3 | 96.3 | 89.8 | 19.5 | 17.5 | 83.3 | 94.4 | 88.9 | 21.5 | 19.3 |
| Top 5 | 83.3 | 96.3 | 89.8 | 20.2 | 18.1 | 75.0 | 96.3 | 85.6 | 21.6 | 19.3 |
| Top 4 | 58.3 | 94.4 | 76.4 | 20.7 | 18.5 | 58.3 | 94.4 | 76.4 | 22.0 | 19.7 |
| Top 3 | 66.7 | 94.4 | 80.6 | 20.5 | 18.4 | 66.7 | 94.4 | 80.6 | 21.8 | 19.6 |
| Top 2 | 66.7 | 98.1 | 82.4 | 21.6 | 19.4 | 66.7 | 98.1 | 82.4 | 22.8 | 20.4 |
| Top 1 | 75.0 | 79.6 | 77.3 | 22.1 | 19.8 | 66.7 | 79.6 | 73.1 | 22.8 | 20.4 |
